# Modeling Contaminant Microbes in Rivers During Both Baseflow and Stormflow

**DOI:** 10.1029/2021GL096514

**Published:** 2022-04-18

**Authors:** J. D. Drummond, T. Aquino, R. J. Davies‐Colley, R. Stott, S. Krause

**Affiliations:** ^1^ University of Birmingham School of Geography, Earth & Environmental Sciences Birmingham UK; ^2^ Université de Rennes CNRS Géosciences Rennes, UMR 6118 Rennes France; ^3^ NIWA (National Institute of Water & Atmospheric Research Ltd.) Hamilton New Zealand; ^4^ Université de Lyon Université Claude Bernard Lyon 1 CNRS ENTPE UMR5023 Ecologie des Hydrosystèmes Naturels et Anthropisés (LEHNA) Villeurbanne France

**Keywords:** hyporheic, immobilization, remobilization, contaminant microbes, particle tracking model, mobile immobile model

## Abstract

Rivers transport contaminant microorganisms (including fecal indicator bacteria and human pathogens) long distances downstream of diffuse and point sources, posing a human health risk. We present a mobile‐immobile model that incorporates transport as well as immobilization and remobilization of contaminant microbes and other fine particles during baseflow and stormflow. During baseflow conditions, hyporheic exchange flow causes particles to accumulate in streambed sediments. Remobilization of stored particles from streambed sediments occurs slowly during baseflow via hyporheic exchange flow, while remobilization is vastly increased during stormflow. Model predictions are compared to observations over a range of artificial and natural flood events in the dairy contaminated Topehaehae Stream, New Zealand. The model outputs closely matched timing and magnitude of *E. coli* and turbidity observations through multiple high‐flow events. By accounting for both state‐of‐flow and hyporheic exchange processes, the model provides a valuable framework for predicting particle and contaminant microbe behavior in streams.

## Key Points

- Remobilization of microbes is dependent on both their antecedent accumulation in streambed sediments and the magnitude of stream flow
- Storm events mobilize microbes from streambed stores that are replenished during the falling limb of the storm hydrograph and baseflow
- Our particle tracking mobile‐immobile model captures exchanges between the surface water, the hyporheic zone and deeper streambed

## 1 Introduction

Public health risks from the presence of contaminant microorganisms in waters, such as human pathogenic bacteria, parasites, or viruses, are a global concern (Ramirez‐Castillo et al., 2015). Although rivers can transport microorganisms to long distances, timescales of retention and persistence in streambed sediments prior to downstream transport can range from days to years (Haggerty et al., 2002; Jamieson et al., 2004; Petersen & Hubbart, 2020), extending potential risks to long timescales after initial contamination of the stream. Stormflow events are known to resuspend retained microbes (Davies‐Colley et al., 2008; McKergow & Davies‐Colley, 2010) with the movement of microbes hypothesized to be linked to bed‐mobilizing flows that remobilize sediments and attached microbes (Cho et al., 2010; de Brauwere et al., 2014; Zhou et al., 2017). However, microbes are also remobilized during steady‐state baseflow (i.e., subcritical flow conditions) below the bed‐mobilizing threshold (Bradshaw et al., 2016; Fluke et al., 2019; Muirhead & Meenken, 2018; Park et al., 2017), therefore providing evidence of other co‐occurring processes that lead to measurable concentrations of microbes in streams during baseflow. Hence, appropriately characterizing transport and retention of contaminant microbes during both baseflow and stormflow conditions is required for predicting in‐stream contamination and assessing microbial hazards.

Hyporheic exchange flow, the transport of solutes, and fine particles, including microbes, to and from the water column via flowpaths through streambed sediments (Boano et al., 2014; Haggerty et al., 2002; Krause et al., 2011, 2017), is an important process, often not considered in models of contaminant microbe behavior in streams. For example, J. D. Drummond et al. (2018) demonstrated that hyporheic exchange flow can cause up to 66% of contaminant microbe inputs into an agriculturally impacted stream to persist for years under baseflow conditions. In fact, the hyporheic zone is an important ecotone for a diverse set of processes that provide opportunities for the self‐purification of rivers, including the storage and degradation of pollutants and modulation of metabolic stream processes (Lewandowski et al., 2019). Turbulence near to the surface water‐sediment interface and advective transport pathways caused by pressure variations at the streambed surface are the main reasons for the exchange of microbes between surface water and streambed sediments and other transient storage areas (Roche et al., 2019) although there are a wide range of hydrostatic and hydrodynamic forces considered as hyporheic exchange processes (Boano et al., 2014; Grant et al., 2011). However, current models used to predict water quality in freshwaters normally assume that microbes can only be transported into streambed sediments by incorporation into aggregates that settle by gravity (e.g., see review by Cho et al., 2016). Hyporheic exchange processes can furthermore result in baseflow remobilization of microbes (J. D. Drummond et al., 2015, 2018) although models that incorporate both baseflow and stormflow attribute baseflow remobilization to other processes, such as biofilm sloughing (Kim et al., 2017; Park et al., 2017).

Available modeling frameworks do not account for baseflow and stormflow fine particle transport, including contaminant microbes, that simulate hyporheic exchange, immobilization, and remobilization processes. A suitable model should be parsimonious, that is, use as few parameters as possible to characterize the key processes and match the data so as to narrow the available parameter space and provide confidence in the best‐fit values (J. Drummond et al., 2019; Kelleher et al., 2019). An appropriate model framework needs to address not only fine particle mobilization and transport during storm events, but also differing transport mechanisms between the rising and falling limb of a storm hydrograph. As it is not yet possible to measure the transport of particles at this level of detail during a storm event, there is scope for model‐based assessments to describe transport behavior of particles over events. During a storm event, particles retained within the hyporheic zone are partially remobilized (J. D. Drummond et al., 2017; Filoso et al., 2015; Harvey et al., 2012; Larsen et al., 2015). During the rising limb stormflow hydrograph, there is net remobilization of retained contaminant microbes (Lamba et al., 2015). Building on this field evidence, we hypothesize that although deposition into the hyporheic zone takes place during the rising limb of the storm event, deposited particles will follow advective porewater paths back into the water column instead of transporting deeper into the streambed. Moreover, we hypothesize that on the falling limb, fine particles are transported as they were during baseflow conditions, where deposition into the hyporheic zone, transport into the deeper streambed, and remobilization back to the water column are all taking place simultaneously. Finally, we aim to explore here how baseflow remobilization occurs not merely after the critical threshold for mobilizing streambed sediments is exceeded, but also because of hyporheic exchange processes combined with the increased remobilization observed during a storm event.

To test the above hypotheses, we developed and validated a particle tracking mobile‐immobile model for in‐stream transport, immobilization, and remobilization of contaminant microbes during both baseflow and stormflow conditions. This new model framework builds on the mobile‐immobile approach (Haggerty & Gorelick, 1995; van Genuchten & Wierenga, 1976) and incorporates hyporheic exchange processes to simulate particles in surface water, the hyporheic region, and the deeper streambed. We aim to capture both the sharp rising limb and slower falling limb of contaminant microbes over storm hydrographs and test our hypotheses on the controlling mechanisms of microbial transport under baseflow and stormflow within a single model framework. We apply this model to in‐stream *E. coli* and turbidity data for a dairy‐contaminated stream in response to (a) a triplet of engineered flood pulses at 1‐day intervals and (b) a two‐peaked natural stormflow event. We demonstrate the ability to capture transport during the sharp rising limb and slower falling limb during both flashy and subdued storm hydrographs in a single model framework. Representing the hyporheic zone as the regulator of simultaneous immobilization and remobilization processes allows both (strongly contrasting) baseflow and stormflow dynamics to be represented within the same model framework for contaminant microbes in streams. We expect that this approach will permit improved predictions of pathogen fate and subsequent risk assessment of disease transmission via freshwaters.

## 2 Materials and Methods

### 2.1 Model Conceptualization

**Figure 1:**
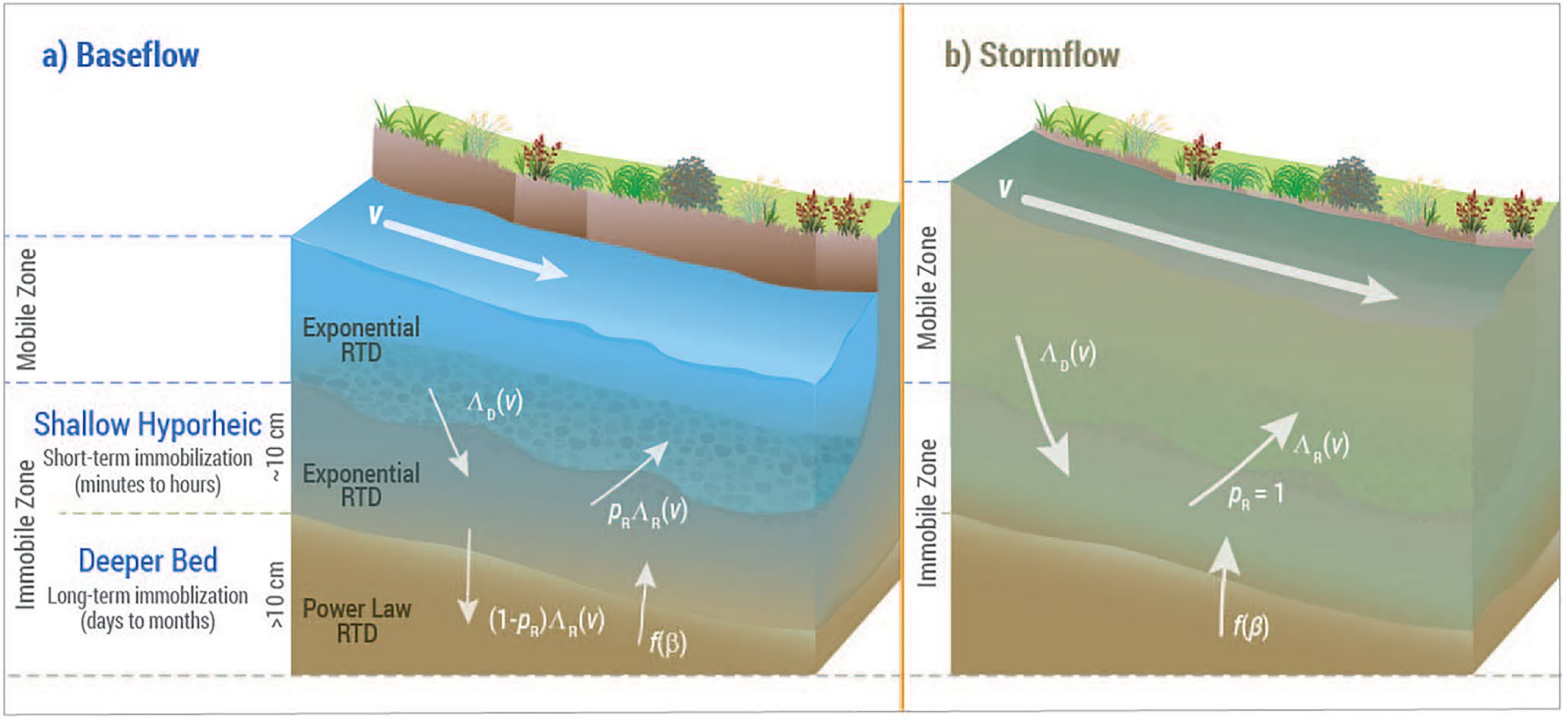
Conceptualization of the particle tracking model for transport, immobilization, and remobilization of contaminant microbes in streams during (a) baseflow and (b) stormflow. Parameters are defined in Table 1. RTD = Residence Time Distribution.

During downstream transport, particles exchange between the mobile and immobile zones within the stream as depicted in Figure 1. The water column represents the mobile zone, while the immobile zone includes both the shallow hyporheic region of the streambed sediment (∼10 cm depth) and the deeper streambed (≳10 cm depth). The actual depth of each of these regions depends on the local hydrogeomorphologic conditions. The key input parameters that have been varied for fitting within the model framework are listed in Table 1 together with specified value ranges.

**Table 1:** Model Variables' Descriptions and Ranges

Description of model variables	Unit	Range
*v*(*t*), Velocity	m s^−1^	From input data, Discharge/(water width × depth)
*Λ*_*D*_, Exchange rate from the water column to hyporheic zone = *c*_*D*_ *v* ^2^; *c*_*D*_ is a deposition coefficient	s^−1^	*c*_*D*_ range: 1 · 10^−5^ − 1 · 10^2^ s m^−2^
*p*_*R*_, Probability of particle remobilizing to the water column versus being transported to the streambed. Being transported to the streambed has probability 1 − *p*_*R*_	dim.	0− 1 during baseflow. During stormflow, *p*_*R*_ = 1 (particles can only return to the water column)
*Λ*_*R*_, Remobilization rate from the hyporheic zone = *c*_*R*_ *v* ^2^; *c*_*R*_ is a remobilization coefficient	s^−1^	*c*_*R*_ range: 1 · 10^−5^ − 1 · 10^2^ s m^−2^
*β*, Power law exponent of the residence time distribution in streambed, controls particle release back to the hyporheic zone	dim.	0–1

*Note.*Inputs are varied following a Monte Carlo approach (Section 2.2). Other fixed input model parameters are described in Text S1 in Supporting Information S1.

#### 2.1.1 Mobile Zone

The mobile zone of the model framework is parameterized by in‐stream velocity (*v*, m s^−1^) and the hyporheic exchange rate (*Λ*_*D*_, s^−1^). Velocity is calculated from measured stream flow, water depth, and average water width (Table 1). Therefore, *v* varies with discharge and is assumed constant between measurements. Exchange from the water column to the underlying sediments, termed the hyporheic exchange rate (*Λ*_*D*_, s^−1^), is an important process that leads to the deposition of microbes and other fine particles with low settling velocities (Boano et al., 2014; J. D. Drummond et al., 2020). Residence times in the water column are exponentially distributed with an average exchange rate into the hyporheic zone proportional to the square of in‐stream velocity (Text S1 in Supporting Information S1, Arnon et al., 2013; Packman et al., 2004), calculated as *Λ*_*D*_ = *c*_*D*_ *v* ^2^, where *c*_*D*_ is a deposition coefficient (Table 1).

#### 2.1.2 Immobile Zone

Contaminant microbes transported into the shallow hyporheic region can either transport further into the deeper streambed or return to the water column, controlled by a resuspension probability *p*_*R*_ that can range from 0 to 1 (Table 1). A *p*_*R*_ of 1 signifies that particles can only follow the transport path back to the water column and conversely, a *p*_*R*_ of 0 signifies that particles can only transport into the deeper streambed. Residence times in the hyporheic zone are exponentially distributed with an average exchange rate back to the water column or into the deeper streambed proportional to the square of in‐stream velocity (termed the remobilization rate, *Λ*_*R*_), based on previous observations of fine sediment remobilization from the streambed (Arnon et al., 2013; Cardenas et al., 1995; Cho et al., 2010). The remobilization rate is calculated as *Λ*_*R*_ = *c*_*R*_ *v* ^2^, where *c*_*R*_ is the remobilization coefficient. Here, we do not require that a critical threshold is met before microbes can be remobilized from the hyporheic zone to either the deeper streambed or water column. This lack of a critical threshold is supported by previous laboratory and fieldwork that demonstrate the remobilization of fine particles during baseflow (Bradshaw et al., 2016; J. D. Drummond et al., 2015; Fluke et al., 2019; Muirhead & Meenken, 2018; Park et al., 2017).

The deeper streambed is characterized by a power law residence time distribution (RTD), based on field observations of microbial retention and release from streambed sediments (Aquino et al., 2015; J. D. Drummond, Aubeneau, & Packman, 2014; J. D. Drummond, Davies‐Colley, et al., 2014; Haggerty et al., 2002), and compared to an exponential distribution that allows for a wider range of times when contaminant microbes are released back to the hyporheic zone. As soon as microbes are released from the deeper streambed to the hyporheic zone, they will again be subject to transport to the water column or back to the streambed with a probability *p*_*R*_ and remobilization rate, *Λ*_*R*._

#### 2.1.3 Stormflow

During stormflow, the same transport processes were considered in the model, but we ran three different scenarios to test our hypotheses on how transport of microbes may differ between the rising and falling limbs of the storm hydrograph. We first assessed model outputs without any changes from baseflow parameters (scenario 1), and then only allowed deposited particles in the hyporheic zone to transport back to the water column by setting *p*_*R*_ = 1 (Section 2.1.2, Figure 1b) during both the rising and falling limbs (scenario 2) and only the rising limb (scenario 3) of the storm hydrograph. This adjustment forces retained or deposited microbes already in the hyporheic zone to remobilize back to the water column instead of deeper into the streambed, aligning with field observations (J. D. Drummond et al., 2015, 2017; Filoso et al., 2015; Harvey et al., 2012; Lamba et al., 2015).

### 2.2 In‐Stream Field Studies of Contaminant Microbe Transport Dynamics

Following the fitting procedure outlined in J. Drummond et al., 2019, we performed several simulations (Text S2 in Supporting Information S1) with parameter sets constrained to match the in‐stream measurements of *E. coli* and turbidity during artificial floods (Section 2.2.1) and a natural storm event (Section 2.2.2) in a dairy cow‐impacted stream in New Zealand. The three scenarios for stormflow as described in 2.1.3 were evaluated for the artificial floods and natural storm event *E. coli* data, separately. Then, the best‐fit scenario was used to fit the turbidity data.

#### 2.2.1 Artificial Floods

An experiment with artificial flood pulses was conducted in the Topehaehae Stream (median flow ∼2.6 · 10^2^ L s^−1^) in the Waikato Region, North Island, New Zealand, using water from a potable supply reservoir as the source (Muirhead et al., 2004). The artificial flood pulses were conducted on 3 successive days by opening a release valve over 30 min, keeping it open for 20 min, and closing it over 10 min. Flow increased ∼5–6− fold from 7.7 · 10^2^ to 4.3 · 10^3^ L s^−1^ during each pulse. The water level, turbidity, and *E. coli* were measured at several sites downstream, and we focus on the furthest site 2.5 km downstream from the reservoir. The average stream width of the study reach was 5.8 m. The increase in the water level during the flood event was confined within the channel without any overbank flow, thereby allowing for a focused study on remobilization of fine particles and *E. coli* from in‐channel sources. For more experimental details, see Text S3 in Supporting Information S1.

#### 2.2.2 Natural Storm

A natural storm event occurred in the Topehaehae Stream in September 1999 in response to 900 mm of rainfall falling in two peaks about 3 days apart (Nagels et al., 2002). Autosamples collected over the natural flood event were analyzed for *E. coli* and turbidity by the same methods as for the artificial flood experiments. In response to this precipitation event, stream flow rose 10‐fold from a baseflow of 5.0 · 10^2^ L s^−1^ before the event to 5.0 · 10^3^ L s^−1^ at the first flood peak and 3.5 · 10^3^ L s^−1^ at the second peak. In‐stream measurements of *E. coli* and turbidity during the natural storm event were made downstream and in a reach with shallower depth, increased width, lower slope, and silty‐sand bed as compared to the artificial flood sampling site. Therefore, microbial exchange parameters are expected to contrast between these sampling sites both during baseflow and in response to the storm event.

## 3 Results and Discussion

### 3.1 Model Performance

**Figure 2:**
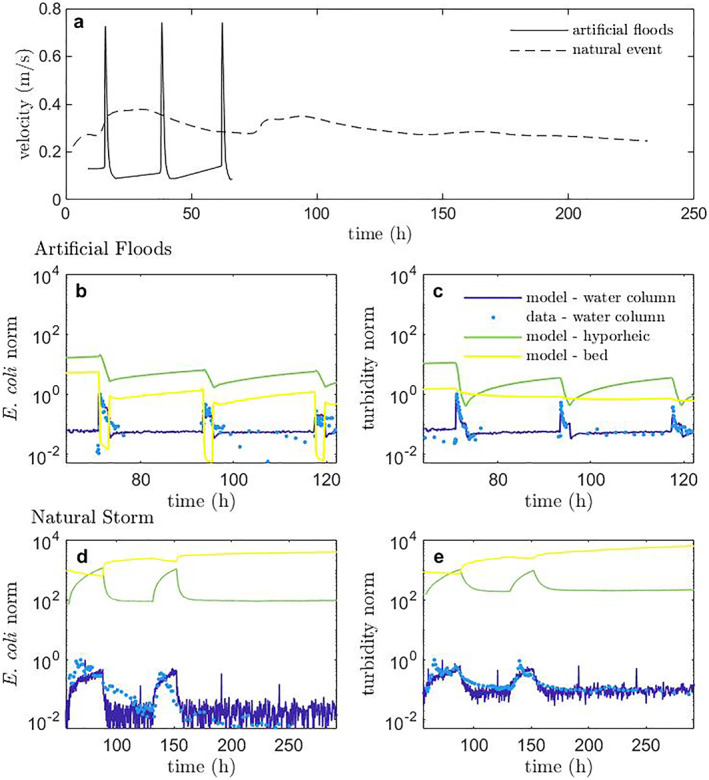
(a) Comparison of velocity during three artificial floods in series to a natural storm event. Topehaehae stream observations and simulations of (b) *E. coli* and (c) turbidity in response to three artificial floods in series and (d) *E. coli* and (e) turbidity in response to a natural storm event. Best‐fit parameters with confidence intervals are shown in Table 2. Norm refers to concentrations normalized by the max concentration in the water column, presented in log_10_ scale. Water column simulations are compared with measured *E. coli* and turbidity data and simulated concentrations in the hyporheic zone (∼10 cm bed sediment depth) and deeper bed (>10 cm depth).

During the artificial floods, the velocity increased 5–6− fold within 30 min, while for the natural flood, the increase was 10− fold but over a much longer time of ∼28 hr (Table 2, Figure 2a). The flow variations between the artificial flood pulses and natural storm event can be explained by (a) the reach sampled during the natural event that has a shallower slope, different streambed sediments and geomorphologic characteristics and (b) the experimental constraints during the artificial floods, which prevented releasing such a large supply of water more slowly but over a longer time period to mimic a natural event. We assessed model performance from Monte Carlo simulations (27 · 10^3^ trials, Text S2 in Supporting Information S1) for *E. coli* for the two types of storm hydrographs (i.e., flashy vs. subdued) for the 3 stormflow scenarios (Section 2.1.3). The best‐fit model, chosen as the lowest model error calculated between the data and model (Text S2 in Supporting Information S1), was found for scenario 2 for the artificial floods (minimum error *θ* = ∼0.14) and scenario 3 for the natural storm (minimum error *θ* = ∼0.23) (Figures 2b, 2c and 2d, 2e, respectively). Best fits and model error assessment are shown for all scenarios for *E. coli* in Figures S1–S6 in Supporting Information S1. The best‐fit scenario was run for turbidity data with minimum error *θ* = ∼0.17 for the artificial floods and *θ* = ∼0.21 for the natural storm events (Figures S7 and S8 in Supporting Information S1, respectively). This result showed that for a flashy event, microbes are resuspended back to the water column during both the rising and falling limb, but only during the rising limb for a subdued natural event. Therefore, during the falling limb of a subdued natural event, deposition both into the deeper bed and resuspension to the water column co‐occur as during baseflow.

**Table 2:** Best‐Fit Parameters, Defined in Table 1, and Associated Confidence Intervals Calculated as ± the Standard Deviation of the Best 0.05% Fits for Water Column *E. coli* and Turbidity Measurements During Three Artificial Flood Events in Series in Topehaehae Stream

Parameter	Artificial floods		Natural storm event	
	*E. coli*	Turbidity	*E. coli*	Turbidity
*Best‐fit model parameters*				
*c*_*D*_ (s m^−2^)	1.7·10^−1^ ± 4.5·10^−2^	1.3·10^−1^ ± 5.1·10^−2^	8.0·10^1^ ± 3.1·10^1^	1.6·10^1^ ± 2.6·10^1^
*c*_*R*_ (s m^−2^)	3.7·10^−3^ ± 1.2·10^−3^	6.7·10^−3^ ± 2.0·10^−3^	1.1·10^−2^ ± 1.6·10^−1^	3.0·10^−3^ ± 4.5·10^−3^
*p*_*R*_	1.1·10^−2^ ± 8.0·10^−3^	1.2·10^−2^ ± 3.6·10^−3^	4.3·10^−1^ ± 1.1·10^−1^	6.5·10^−1^ ± 1.6·10^−1^
*β*	9.9·10^−1^ ± 1.9·10^−1^	6.3·10^−1^ ± 1.4·10^−1^	2.4·10^−1^ ± 1.2·10^−1^	1.6·10^−1^ ± 5.6·10^−2^
*Temporally averaged rates and residence time estimates*				
*Λ*_*D*_ baseflow (s^−1^)	2.9·10^−3^	2.2·10^−3^	3.9·10^0^	7.6·10^−1^
*Λ*_*D*_ peak stormflow (s^−1^)	9.3·10^−2^	7.0·10^−2^	1.1·10^1^	2.3·10^0^
*Λ*_*R*_ baseflow (s^−1^)	6.3·10^−5^	1.1·10^−4^	5.3·10^−4^	1.5·10^−4^
*Λ*_*R*_ peak stormflow (s^−1^)	2.0·10^−3^	3.7·10^−3^	1.6·10^−3^	4.3·10^−4^
Water column residence time baseflow (1/*Λ*_*D*_, hour)	1.0·10^−1^	1.3·10^−1^	7.1·10^−5^	3.7·10^−4^
Water column residence time stormflow (1/*Λ*_*D*_, hour)	3.0·10^−3^	4.0·10^−3^	2.4·10^−5^	1.2·10^−4^
Hyporheic residence time baseflow (1/*Λ*_*R*_, hour)	4.4·10^0^	2.5·10^0^	5.2·10^−1^	1.9·10^0^
Hyporheic residence time peak stormflow (1/*Λ*_*R*_, hour)	1.4·10^−1^	7.6·10^−2^	1.7·10^−1^	6.4·10^−1^

*Note*. Rates and residence time estimates were calculated based on average best‐fit model parameters.

### 3.2 Rates of Exchange and Residence Times in Water Column, Hyporheic, and Streambed Regions During Baseflow and Stormflow

Our modeling results advance towards a mechanistic understanding of high variation in *E. coli* concentrations during baseflow, even over relatively short timescales (Muirhead & Meenken, 2018). Both *E. coli* and turbidity varied strongly in response to small perturbations in flow (Figure 2). The observed variability is dependent on the timescales of storage and exchange between the water column, hyporheic zone, and deeper bed, explaining why sometimes there is net deposition and other times net erosion/remobilization during storm events (Lamba et al., 2015). The continued release of *E. coli* from sediments immediately following a storm event after baseflow conditions return has been observed and associated with biofilm sloughing or other mechanisms (Kim et al., 2017; Park et al., 2017; Yakirevich et al., 2013). However, our model framework suggests that this can be more simply explained by hyporheic exchange, not only into, but also out of, the streambed, depending on flow conditions.

The model matched the experimentally observed decrease in peak concentration with each subsequent artificial flood pulse, representing the depletion of *E. coli* and fine sediment (i.e., turbidity) from the streambed sediments (Figures 2b and 2c, respectively). In general, best‐fit parameters for the artificial floods were very similar for *E. coli* and turbidity (Table 2). Overall, all model parameters fell within the expected ranges. Specifically, the hyporheic exchange rates were 2.9 · 10^−3^ and 2.2 · 10^−3^ s^−1^ for *E. coli* and turbidity, respectively (Table 2), falling within the range of previously reported values (Cheong et al., 2007; J. D. Drummond et al., 2020). Since advective exchange of water and turbulence at the surface water‐sediment bed interface controls the transport of contaminant microbes and other fine particles into the hyporheic region (Boano et al., 2014; J. D. Drummond et al., 2015), the finding that the hyporheic exchange rates are similar confers confidence in the model framework in that it is capable of appropriately characterizing this important process. Residence times in the hyporheic zone ranged from 2.5–4.4 hr during baseflow (Table 2) and decreased to less than an hour during the flood release events. The model simulations demonstrate that the exchange and retention of contaminant microbes still occur during storm flow even for events as flashy and extreme as in the artificial flood experiment. In fact, exchange rates into the hyporheic zone actually *increase* with the increased stream flow velocity during the flood event but with lower retention times in the hyporheic region before being released back to the water column (Table 2).

During baseflow, microbes and fine sediments were mainly transported from the hyporheic zone into the streambed and not immediately back to the water column as shown by a very low *p*_*R*_ of ∼1 · 10^−2^ for both *E. coli* and turbidity (Table 2). A low *p*_*R*_ aligns with previous observations of microbial transport during baseflow, using a model that assumed microbes that transported into the sediments were slowly released back to the water column, following a power law RTD (e.g., J. D. Drummond, Aubeneau, & Packman, 2014; J. D. Drummond, Davies‐Colley, et al., 2014). Moreover, the remobilization rate (*Λ*_*R*_) was lower than the deposition rate into the hyporheic zone (*Λ*_*D*_) (Table 2) as expected based on immobilization processes in the hyporheic zone (Boano et al., 2014; J. D. Drummond, Aubeneau, & Packman, 2014; J. D. Drummond, Davies‐Colley, et al., 2014). Therefore, during baseflow, both *E. coli* and fine sediments transport into the hyporheic region and within hours also into the deeper streambed, where retention times are longer and release back into the hyporheic zone is slow and can take hours to months (J. D. Drummond et al., 2018; J. Drummond et al., 2019). One difference between the measured microbes and fine sediments (turbidity) was a slightly lower power law slope in the deeper streambed, *β*, for *E. coli* than turbidity, suggesting increased retention and slower release of *E. coli* back to the hyporheic zone. A lower *β* for *E. coli* can either be explained by the increased attachment of microbes that excrete extracellular polymeric substances, which could decrease their release from the deeper streambed to the hyporheic region and eventually back to the water column (Battin et al., 2016; Eboigbodin & Biggs, 2008) or alternatively, a result of the long‐term inactivation of *E. coli* in the streambed.

Higher *E. coli* and turbidity values in the hyporheic and deeper streambed regions were obtained in simulations than in the water column (Figures 2b–2e), matching field observations. However, the interplay between the three model regions differs between *E. coli* and turbidity (Figures 2b and 2c, respectively) even with small differences in parameter values. Overall, we observed that unsurprisingly, the hyporheic zone is much more dynamic than the more stable deeper bed, exhibiting sharper changes in concentration during the flood events. The new model framework matches the artificial flood data and demonstrates how the hyporheic zone connects the surface water with the deeper bed and regulates the slow release of contaminant microbes back into the water column during baseflow and fast release during stormflow, appropriately representing the transport and accumulation behaviors of both microbes and fine particles.

Our work supports the concept that remobilization of *E. coli* from the sediment bed during natural storm events only leads to partial removal as has been observed experimentally (J. D. Drummond, Aubeneau, & Packman, 2014; J. D. Drummond, Davies‐Colley, et al., 2014; Stocker et al., 2018). We were able to provide some insight into microbial release during a natural storm event and deposition co‐occurring with remobilization during the falling limb—something we have not been able to assess experimentally. However, we do not assess the parameter values in detail for the natural storm event since this event could have also included inputs from storm runoff into the stream, which was not measured, while the artificial floods caused remobilization only from the bed. Significant amounts of *E. coli* can wash into streams with surface runoff water during storm events (Boithias et al., 2021) with in‐stream concentrations linked to land use (Bradshaw et al., 2016; Pandey et al., 2018). Surface runoff during storm events likely explains the gradual decrease in *E. coli* concentrations during the falling limb as compared to the sharper decrease in model output concentrations (Figure 2). In general, by only including hyporheic exchange flow and release of contaminant microbes from the streambed during stormflow, we were able to represent the variation in concentrations often observed in streams under dynamic flow and advance toward predicting how microbes are transported between the zones (i.e., surface water, hyporheic, and streambed).

## 4 Conclusions

Our new model framework for fine particle and contaminant microbe transport, hyporheic exchange flow, immobilization, and remobilization during both baseflow and stormflow was able to represent both a series of three artificial floods and a two‐peak natural storm event. The model captures the dynamic transport between stream zones with quick exchange into and out of the hyporheic region and slow release from the streambed, so contributing to mechanistic understanding of contaminant microbe accumulation patterns in streams under variable flow conditions. Natural variation in microbe concentrations during baseflow and stormflow can be represented by this model framework and differential deposition and resuspension during flashy versus subdued storm hydrographs. Future applications of this model to storms in series, accounting for legacy effects from previous storms and the replenishment of microbes in the sediments between events, should further improve characterization of contaminant microbe behavior during both baseflow and stormflow. This should, in turn, assist with assessing waterborne microbial hazards.

## Conflict of Interest

The authors declare no conflicts of interest relevant to this study.

## Supporting information

Supporting Information S1

## Data Availability

The model was implemented in the C++ programming language and is available under an open‐source license on Github (https://github.com/tcAquino/EColi). The specific version used in this work is available on Zenodo (https://doi.org/10.5281/zenodo.5095334).
