# Supplementary material for: Modeling Contaminant Microbes in Rivers During Both Baseflow and Stormflow
Source: Geophys Res Lett. 2022 Apr 18;49(8):e2021GL096514. doi: 10.1029/2021GL096514 (PMC9286818; doi:10.1029/2021GL096514)
Supplement: Supplementary file 1 — Supporting Information S1 [file GRL-49-0-s001.pdf]

Geophysical Research Letters

Supporting Information for

Modeling Contaminant Microbes in Rivers During Both Baseflow and Stormflow

J. D. Drummond[1*], T. Aquino[2], R. J. Davies-Colley[3], R. Stott[3], and S. Krause[1,4]

[1] University of Birmingham, School of Geography, Earth & Environmental Sciences, Edgbaston, Birmingham,

[2] Univ. Rennes, CNRS, Géosciences Rennes, UMR 6118, 35000 Rennes, France.

[3] NIWA (National Institute of Water & Atmospheric Research Ltd.), Hamilton, New Zealand.

[4] Univ Lyon, Université Claude Bernard Lyon 1, CNRS, ENTPE, UMR5023, Ecologie des Hydrosystèmes Naturels et Anthropisés (LEHNA), 69622, Villeurbanne, France

Contents of this file

Text S1 to S3
Figures S1 to S8

Introduction

The supporting information includes an extended version of the model description (Text S1) and simulations to assess model performance (Text S2), and additional information on the experimental data (Text S3). Model performance was assessed from Monte Carlo simulations ($27 \cdot 10^3$ trials) for *E. coli* in both the artificial floods in series and natural storm event for the three storm flow scenarios: 1) same parameters as baseflow, 2) $p_R = 1$ for both rising and falling limb of storm hydrograph, and 3) $p_R = 1$ for both rising and falling limb of storm hydrograph.
Figures S1-S6 show the model error vs. parameter and best-fit model outputs plotted against data for the 3 scenarios for *E. coli* measured during the artificial floods in series and the natural storm event. Figures S7 and S8 show the model error vs. parameter and best-fit model outputs plotted against data for turbidity measurements using the scenario that was the best-fit for *E. coli* for the artificial floods in series and natural storm, respectively.

Text S1. Particle tracking mobile-immobile model description

We have developed and implemented a particle tracking approach to model transport, retention, and remobilization in river and stream reaches, both under baseflow and stormflow conditions. In our model, transport of *E. coli*, fine sediments, or other solutes is represented by discretizing the transported quantity into a set of Lagrangian particles. Note that these particles do not usually represent the number of physical particles, such as *E. coli* cells, as this would be too computationally expensive. Rather, each particle represents a discretized portion of the total transported mass (Sund et al., 2019). Transport of the Lagrangian particles along the downstream direction is then modeled as a continuous time random walk (Berkowitz et al., 2006). Particles undergo successive steps, each characterized by a downstream displacement length and duration. The downstream position X_n of a particle upon completion of the nth step, along with the corresponding arrival time T_n and mass carried M_n, obey the stochastic recursion relations

$$X_{n+1} = X_n + \Delta X_n^{Z_n}, \quad (1a)$$

$$T_{n+1} = T_n + \Delta T_n^{Z_n}, \quad (1b)$$

$$M_{n+1} = M_n e^{-\lambda^{Z_n} \Delta T_n^{Z_n}}. \quad (1c)$$

We now provide a brief overview of these quantities; the transport and retention processes in each of these three zones are discussed in detail below. The displacements $\Delta X_n^{Z_n}$ and step durations $\Delta T_n^{Z_n}$ depend on the zone Z_n the particle is currently in. The model considers three vertical regions, or zones: the water column, $Z_n = W$; the hyporheic zone, $Z_n = H$; and the deep streambed, $Z_n = B$. Note that particles remain in the same zone for the duration of each step. The next zone visited, Z_{n+1}, depends on the current zone Z_n in a potentially stochastic manner, as explained in more detail below. The water column displacement ΔX_n^W is determined by flow velocity and water column residence times. We take downstream flow velocities in the hyporheic zone and streambed and streambed to be negligible in comparison to the water column, so that $\Delta X_n^H = \Delta X_n^B = 0$ in the remaining two regions. Thus, our approach is of the mobile-immobile type (Haggerty & Gorelick, 1995; Van Genuchten & Wierenga, 1976). In addition, we expect longitudinal (i.e., along the downstream direction) hydrodynamic plume dispersion to be dominated by the effect of retention in the immobile zones. As such, we neglect longitudinal diffusion, as well as the dispersion effects arising from vertical variability in flow within the water column, where we consider a depth-averaged flow velocity. The mass of a particle can decay at a region-dependent rate, due, e.g., to cell death or inactivation. We assume no net growth or decay in the water column and hyporheic zone, $\lambda^W = \lambda^H = 0$. Mass decay in the bed was assumed to be due to inactivation and the corresponding rate was set to $\lambda^B = 1.4 \cdot 10^{-6} \mathrm{s}^{-1}$ (Drummond et al., 2014, Sinton et al., 2002). Note that net growth rates (e.g., due to cell division) can also be modeled by taking the rates to be negative.

From the positions X_n, masses M_n, and the corresponding arrival times T_n, computed according to Eqs. (1), the positions at arbitrary times t can be obtained by interpolation. For a given particle, an arbitrary time t will fall between some two arrival

times, $T_n \leq t < T_{n+1}$. The particle will be in some zone Z_n, and the corresponding masses are obtained as

$$M_p(t) = M_n e^{-\lambda^{Z_n}(t-T_n)}. \tag{2}$$

In the simulations performed here, particle masses are computed at a set of 1000 logarithmically-spaced times of interest. Masses in each region are then obtained by summing over the interpolated masses of all particles found within at the given time. Boundary conditions and the associated initial conditions for X_0 and T_0 are discussed below. We note that particle positions can be similarly interpolated if needed.

The subdivision of the system into three vertical zones with different properties in order to account for retention has been previously employed (Aquino et al., 2015; Drummond et al., 2012; Li et al., 2019). However, to the best of our knowledge, we provide the first model of this type accounting for flow-velocity-dependent deposition and resuspension, as discussed in more detail in the remainder of this section.

Water column

As mentioned above, transport in the water column is assumed to be advective, according to the depth-averaged flow velocity $v(t)$, which depends on time t. While a theoretical model could also be employed, here the flow velocity is parameterized from experimental data as follows. The water column heights $h(t_i)$ were measured nearby the sampling site and converted to the flow rate $q(t)$ (details in experimental section Text S3), at a set of $n_f =$ times $t = t_i$, $i = 1, \dots, n_f$. The reach averaged width w of the stream measured along the reach was used to calculate the flow velocity at each measurement time as

$$v(t_i) = \frac{q(t_i)}{h(t_i)w}. \tag{3}$$

For the purpose of the model, the measurement times t_i can be arbitrary, and in particular need not be evenly spaced in time. Between two measurements t_i and t_{i+1}, we approximate the flow velocity as remaining constant and equal to $v(t_i)$. We assume also that the flow velocity is the same across the domain of interest, and changes propagates instantly across it.

We now turn to the step durations ΔT_n^W, which are related to both velocity variability and the residence times in the water column. We model the retention time (RT) probability density function (PDF) $\phi_W(\cdot\,|v)$, conditioned on current water column velocity v, as exponential with a deposition rate $\Lambda_D(v)$,

$$\phi_W(\tau|v) = \Lambda_D(v)e^{-\Lambda_D(v)\tau}. \tag{4}$$

That is, for a given velocity v, the probability of a residence time between τ and $\tau + d\tau$ during a visit to the water column is given by $\phi_W(\tau|v)\,\mathrm{d}\tau$. While any form of $\Lambda_D(v)$ is allowed, we focus here, as discussed in the main text, on a quadratic dependency on velocity,

$$\Lambda_D(v) = c_D v^2, \tag{5}$$

with a single fitting coefficient c_D.

The use of an exponential distribution corresponds to the approximation of a constant rate of deposition for a given velocity. This approximation is appropriate for processes that do not have extremely broad (i.e., infinite mean or variance, see, e.g., Feller, 1966) distributions of waiting times, when modeling transport on time scales on the order of the average residence time $1/\Lambda_D(v)$ or larger. The practical advantage lies in the fact that the exponential PDF characterizes the waiting times of a Poisson process, which is the only memoryless process (Van Kampen, 1981). This means that the distribution of remaining waiting times given that a particle has been waiting for some amount of time is again the same exponential distribution (i.e., particles have no memory of how long they have been within the current zone).

Consider a given step n where the particle is in the water column, $Z_n = W$. Let the time T_n at the beginning of the step lie between the flow measurement times t_i and t_{i+1}. Then, generate a residence time τ_n according to the exponential distribution of Eq. (4) with velocity $v = v(t_i)$. We set

$$\Delta T_n^{Z_n} = \min\{\tau_n, t_{i+1} - t_n\}, \tag{6}$$

that is, the current step duration is τ_n if the time of the next change in velocity is not reached, or the time until the next change in velocity otherwise. If the time of the next change in velocity is reached before deposition occurs, the particle stays in the water column and next zone is $Z_{n+1} = Z_n = W$. Otherwise, the particle is deposited into the hyporheic zone, and $Z_{n+1} = H$. In either case, the downstream step length is determined according to the current velocity as:

$$\Delta X_n^{Z_n} = v(t_i)\Delta T_n^{Z_n}. \tag{7}$$

Because of the lack of memory of the exponential distribution, if no transition occurs, the velocity can then be updated and the procedure repeated until a desired time or distance is reached, or deposition occurs. This incurs no further approximations.

Hyporheic zone

Remobilization in the hyporheic zone is modeled through an approach similar to that used for deposition from the water column. In this case, remobilization is associated with an RTPDF:

$$\phi_H(\tau|v) = \Lambda_R(v)e^{-\Lambda_R(v)\tau}, \tag{8}$$

again exponential and conditioned on the current water column velocity v. Note that, while we neglect downstream advection in the hyporheic zone, which is typically much slower than in the water column, we consider that water column flow velocities influence remobilization rates, as discussed in the main text. While the resuspension rate $\Lambda_R(v)$ can again be chosen arbitrarily, we employ a quadratic relationship as for the deposition rate,

$$\Lambda_R(v) = c_R v^2, \tag{9}$$

with a single (different) fitting coefficient c_R. The procedure for determining step durations ΔT_n^H is the same as for the water column, but no downstream displacement occurs, i.e., $\Delta X_n^H = 0$.

The key difference compared to the water column dynamics is that particles may be either resuspended into the water column, or deposited further into the deep streambed. Thus, once a transition occurs, at the end of some step n, we have $Z_{n+1} = W$ with a resuspension probability p_R, and $Z_{n+1} = B$ otherwise. As discussed in the main text, different scenarios were investigated for the probability p_R; in the final model results, it was considered as a fitting parameter under normal flow conditions, and set to unity (certain resuspension) during storm surge events. The three scenarios assessed here were 1) same parameters as baseflow, 2) $p_R = 1$ for both rising and falling limb of storm hydrograph, and 3) $p_R = 1$ for both rising and falling limb of storm hydrograph, and 3) $p_R = 1$ for only rising limb of storm hydrograph. We note that the model is flexible, and different deposition and remobilization rates $\Lambda_D(v)$ and $\Lambda_R(v)$ may also be considered during base flow and storm surge, although this was not done here.

Deep streambed

Retention times in the deep streambed below the hyporheic zone are known to be extremely broadly-distributed (Drummond et al., 2018; Haggerty et al., 2002; Jamieson et al., 2004; Petersen & Hubbart, 2020). Disregarding the long-term effect of the finite depth of the bed (Drummond et al., 2012), they can be modeled with recourse to infinite-mean distributions; here, we employ a skewed Lévy-stable distribution of exponent $0 < \beta$ <1 (Feller, 1966). This type of distribution generalizes the Gaussian distributions arising from the classical central limit theorem to the case of an infinite mean (extremely broad variability) and positive support (waiting times cannot be negative). For example, pure diffusion along one dimension leads to waiting times to reach a one-side boundary that follow this type of distribution with $\beta = 1/2$ (Feller, 1966). The full RTPDF does not in general have an analytical closed form, but the large-time behavior follows a power law,

$$\phi_B(\tau) \propto \frac{1}{t_b}\left(\frac{t}{t_b}\right)^{-1-\beta}. \qquad (10)$$

The characteristic residence time t_b and the exponent β are the two model fitting parameters characterizing the deep streambed. In all scenarios, t_b was set to 1 to simplify fitting based on previous fitting of field particle data (Drummond et al., 2019).

Note that bed residence times are taken not to depend on water column velocity, which is reasonable in the deep streambed. Thus, in this case, the step durations ΔT_n^B simply coincide with the residence times and are generated according to ϕ_B. Remobilization from the streambed always occurs to the hyporheic zone, so that $Z_{n+1} = H$.

Boundary and initial conditions

We discretize the upstream inflow of transported quantities in time steps $\Delta t_{inj} = 100s$. At intervals of Δt_{inj}, the total mass that would be injected during the next time

interval is approximated as an instantaneous pulse of total mass $c_{inj}v(t_i)\Delta t_{inj}$, divided equally amongst 100 Lagrangian particles. Here, t_i is the time at the beginning of the time interval, and c_{inj} is the injection concentration at the upstream boundary in terms of transported mass per unit downstream distance, which is assumed constant. While the model can handle an arbitrary, time-independent upstream concentration, in the absence of detailed data, this simplifying assumption allows us to focus on the role of transport, deposition, and resuspension dynamics, as discussed in the main text. Since transported particles are assumed to be non-interacting amongst themselves, transport can be computed separately for each discretized pulse and the separate pulses linearly superimposed. Furthermore, since results are normalized by the maximum concentration observed in the water column during the time window of interest, linearity allows us to set $c_{inj} = 1$, in arbitrary units. Note also that concentrations normalized by maximum concentration are equivalent to masses normalized by maximum mass.

In order to reduce the computational expense, the model is run in a stream stretch from a position $100\ m$ upstream of the *E. coli* measurement station, defining the initial position X_0 of injected particles. The downstream boundary condition at the measurement station is set as absorbing, since under the assumption of purely advective transport mass cannot flow upstream. In order to obtain a meaningful initial condition, we start from a domain initially free of transported quantities and run the model for $2.0 \cdot 10^5$s at a fixed flow velocity $v(0)$, in order to ensure steady state conditions are reached. Using the resulting initial condition, which defines the initial position X_0 for each particle already in the domain, we then run the full model starting at time $T_0 = 0$, accounting for variable flow together with upstream injection. Particle masses in each region are recorded as discussed above from time $1s$ up to a final time $1.0 \cdot 10^5$s.

Text S2. Simulations to assess model performance

We sampled the parameter space using a Latin Hypercube approach ($27 \cdot 10^3$ trials, e.g., Kelleher et al., 2019). The Balanced mean square error ($\hat{\theta}$) (Bottacin-Busolin et al., 2011) was used to assess performance. This is defined as:

$$\hat{\theta} = \left(\frac{1}{n}\left[\frac{\sum_{i \subset n_A}\left(C_{sim,i}(\theta) - C_{obs,i}\right)^2}{(\max(C_{obs}) - \min(C_{obs}))^2}\right] + \left[\frac{\sum_{i \subset n_B}(\log(C_{sim,i}(\theta)) - \log(C_{obs,i}))^2}{(\max(\log(C_{obs})) - \min(\log(C_{obs})))^2}\right]\right)^{\frac{1}{2}}, (11)$$

where the total number of observations, n, is the sum of n_A and n_B , defined as the number of observations above and below a threshold concentration, respectively. A 20% of the peak threshold concentration has been shown to provide a balanced weight that considers *all* of the breakthrough curve, both the peak and tail (Riml et al., 2013; Bottacin-Busolin et al., 2011). To test whether the parameter values corresponding to the lowest model errors were identifiable (e.g., Kelleher et al., 2013), we plotted model error

against parameter values (e.g., Wagener & Kollat, 2007). First, model performance was assessed for *E. coli* only for all three stormflow scenarios 1) same as baseflow, 2) $p_R = 1$ during the rising and falling limb of the storm hydrograph, 3) $p_R = 1$ only for the rising limb of the storm hydrograph as shown for the artificial flood events (Figure S1-S3) and natural flood event (Figure S4-S6). In each figure model error was plotted against parameter values for the top 1% of the best-fits (i.e., lowest model error) and the best-fit model is presented with the *E. coli* data. We found a narrowing of parameter space with decreased error for all parameters, especially for our best-fit scenarios (Figure S2 and S6). Then, turbidity was fit following the *E. coli* scenario with the lowest model error, which was scenario 2 for the artificial floods (Figure S7) in series and scenario 3 for the natural storm event (Figure S8). Best-fit parameters are presented in the main text (Table 2).

Text S3. Further description of experimental campaigns

Both the artificial flood events in series and natural storm event were recorded in the Topehaehae stream in Waikato, NZ. However, the natural storm event sampling location was ~13.5 km downstream of the artificial floods sampling location. For the artificial floods, the water level was manually recorded by reading staff gauges every ~5 min during the artificial flood releases and every ~1-2 h in between releases (Muirhead et al., 2004). Therefore, the rating curve to convert water depth to flow rate was already established for this site and used for the experimental campaign. For the natural flood event, water levels during this 15-month period were monitored with a Greenspan pressure recorder, and a stage-discharge rating curve was used to estimate flows at times of sampling (Nagels et al., 2002). In these experiments, *E. coli* was measured using the Colilert® Quanti-tray/2000 (IDEXX) multi-well (MPN) method and expressed in colony-forming units (cfu) 100 mL^{-1} as reported in the original publications by Nagels et al. (2002) and Muirhead et al. (2004). However, the modeling normalizes the values and does not report it in terms of microbial units (CFU or MPN). All turbidity measurements (Nephelometric turbidity units, NTU) were made on the *same* laboratory nephelometer, a Hach 2100 calibrated to formazin.

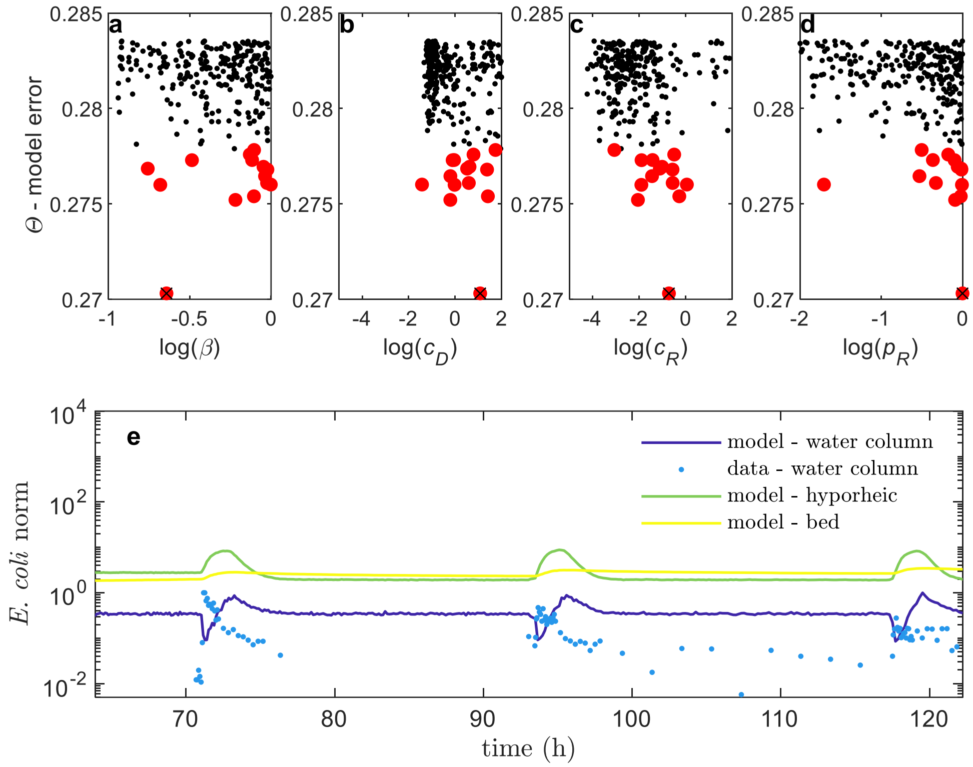

Figure S1. *E. coli* Artificial Floods Scenario 1: stormflow parameters do not change from baseflow parameters. Model error vs. parameter values to demonstrate parameter sensitivity of best-fit model parameters of in-stream measurements of *E. coli* (a-d). Minimum error $\theta = \sim 0.27$. Best-fit model outputs are plotted against data (e). *E. coli* norm = concentrations normalized by the maximum concentration in the water column. Black dots represent parameter values that meet a behavioral error threshold (top 1%) and red dots represent optimal parameter values (top 5% of the 1% shown or 0.05% of the total $27 \cdot 10^3$ runs).

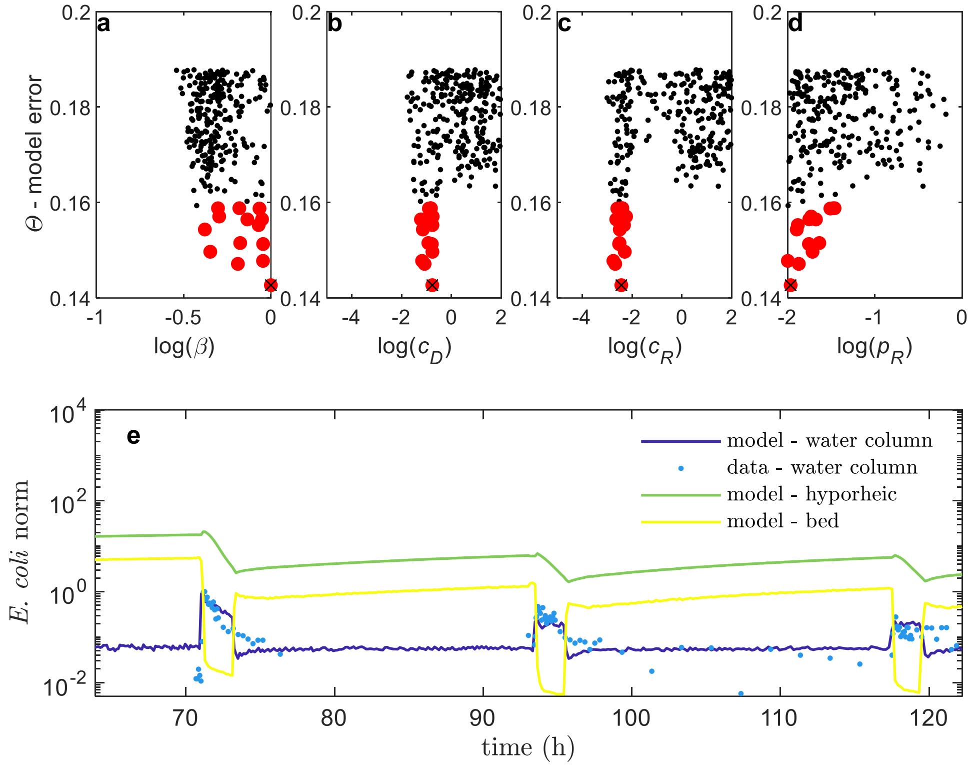

Figure S2. *E. coli* Artificial Floods Scenario 2: during stormflow rising and falling limb deposited particles in the hyporheic zone are only allowed to transport back to the water column by setting $p_R = 1$ (Section 2.1.2, Figure 1B). Model error vs. parameter values to demonstrate parameter sensitivity of best-fit model parameters of in-stream measurements of *E. coli* (a-d). Minimum error $\theta = \sim 0.14$. Best-fit model outputs are plotted against data (e, same as Fig. 2b). *E. coli* norm = concentrations normalized by the maximum concentration in the water column. Black dots represent parameter values that meet a behavioral error threshold (top 1%) and red dots represent optimal parameter values (top 5% of the 1% shown or 0.05% of the total $27 \cdot 10^3$ runs).

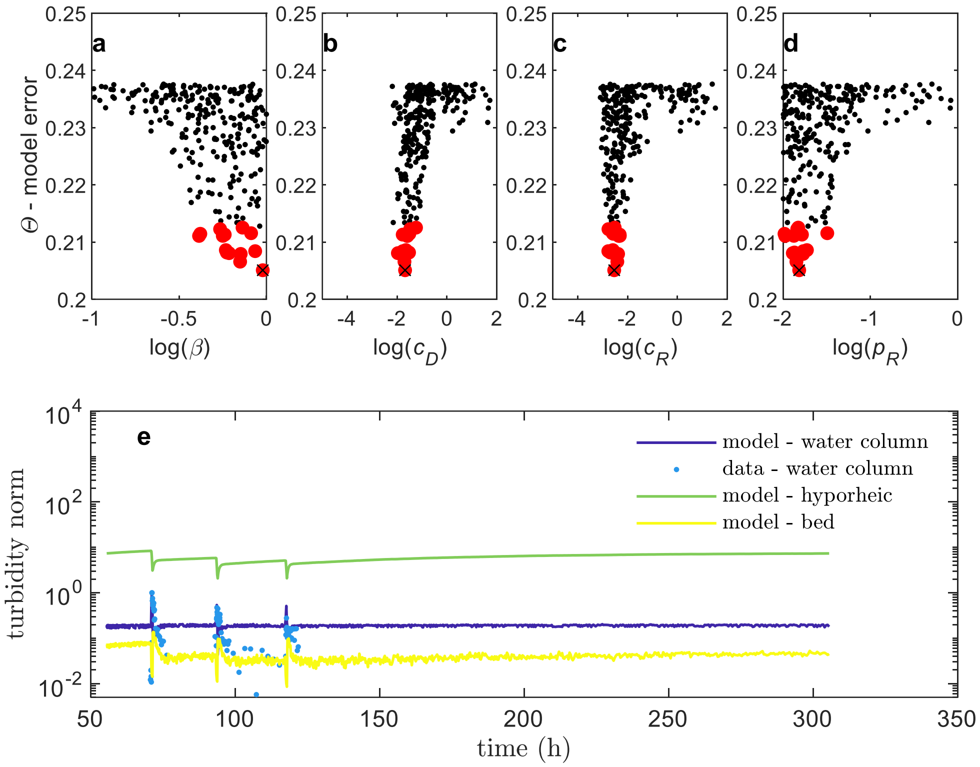

Figure S3. *E. coli* artificial floods Scenario 3: during stormflow rising limb deposited particles in the hyporheic zone are only allowed to transport back to the water column by setting $p_R = 1$ (Section 2.1.2). Model error vs. parameter values to demonstrate parameter sensitivity of best-fit model parameters of in-stream measurements of *E. coli* (a-d). Minimum error $\theta = \sim 0.20$. Best-fit model outputs are plotted against data (e). *E. coli* norm = concentrations normalized by the maximum concentration in the water column. Black dots represent parameter values that meet a behavioral error threshold (top 1%) and red dots represent optimal parameter values (top 5% of the 1% shown or 0.05% of the total $27 \cdot 10^3$ runs).

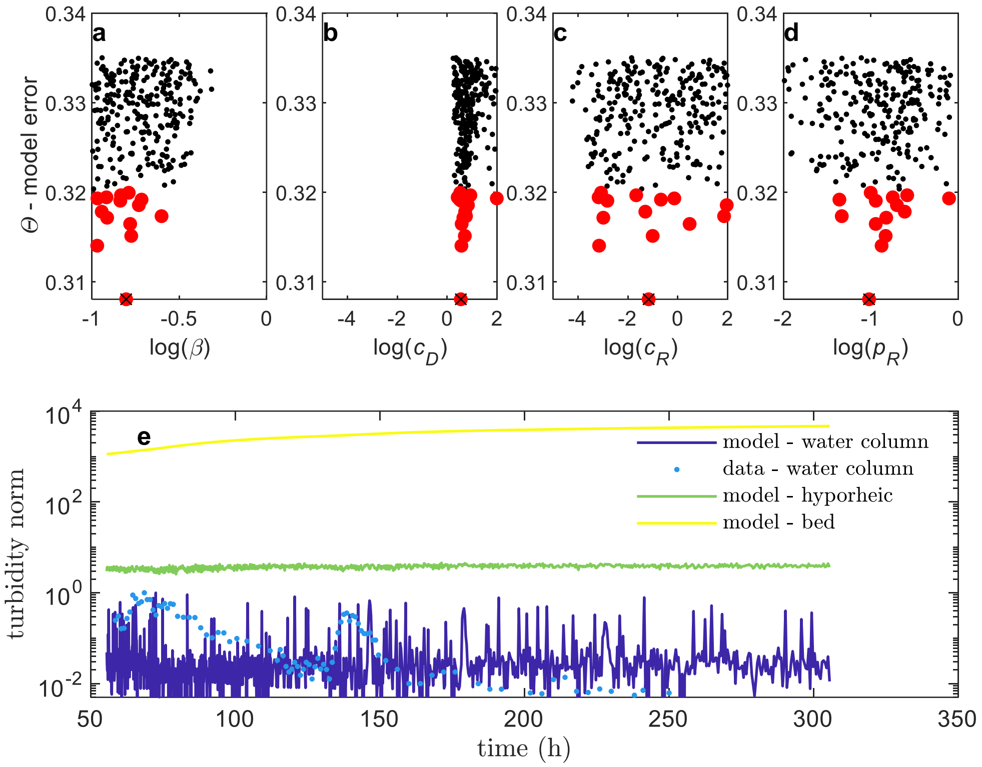

Figure S4. *E. coli* natural storm event Scenario 1: stormflow parameters do not change from baseflow parameters. Model error vs. parameter values to demonstrate parameter sensitivity of best-fit model parameters of in-stream measurements of *E. coli* (a-d). Minimum error $\theta = \sim 0.31$. Best-fit model outputs are plotted against data (e). *E. coli* norm = concentrations normalized by the maximum concentration in the water column. Black dots represent parameter values that meet a behavioral error threshold (top 1%) and red dots represent optimal parameter values (top 5% of the 1% shown or 0.05% of the total $27 \cdot 10^3$ runs).

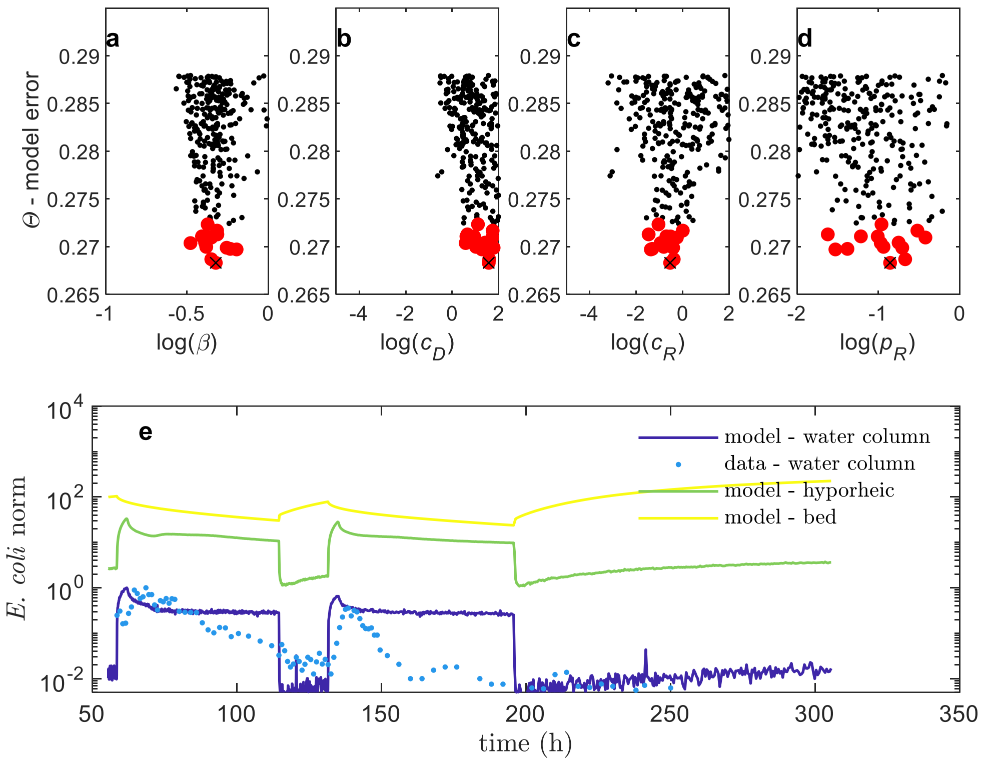

Figure S5. *E. coli* natural storm event Scenario 2: during stormflow rising and falling limb deposited particles in the hyporheic zone are only allowed to transport back to the water column by setting $p_R = 1$ (Section 2.1.2). Model error vs. parameter values to demonstrate parameter sensitivity of best-fit model parameters of in-stream measurements of *E. coli* (a-d). Minimum error $\theta = \sim 0.27$. Best-fit model outputs are plotted against data (e, same as Fig. 2b). *E. coli* norm = concentrations normalized by the maximum concentration in the water column. Black dots represent parameter values that meet a behavioral error threshold (top 1%) and red dots represent optimal parameter values (top 5% of the 1% shown or 0.05% of the total $27 \cdot 10^3$ runs).

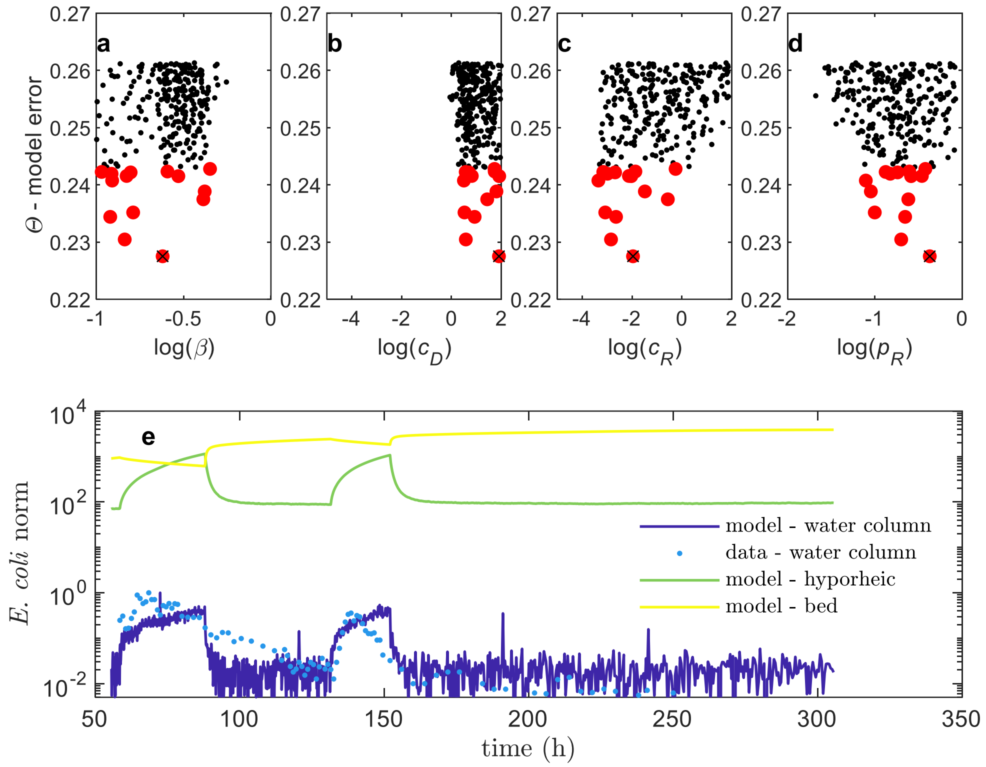

Figure S6. *E. coli* natural storm event Scenario 3: during stormflow rising limb deposited particles in the hyporheic zone are only allowed to transport back to the water column by setting $p_R = 1$ (Section 2.1.2, Figure 1D). Model error vs. parameter values to demonstrate parameter sensitivity of best-fit model parameters of in-stream measurements of *E. coli* (a-d). Minimum error $\theta = \sim 0.23$. Best-fit model outputs are plotted against data (e). *E. coli* norm = concentrations normalized by the maximum concentration in the water column. Black dots represent parameter values that meet a behavioral error threshold (top 1%) and red dots represent optimal parameter values (top 5% of the 1% shown or 0.05% of the total $27 \cdot 10^3$ runs).

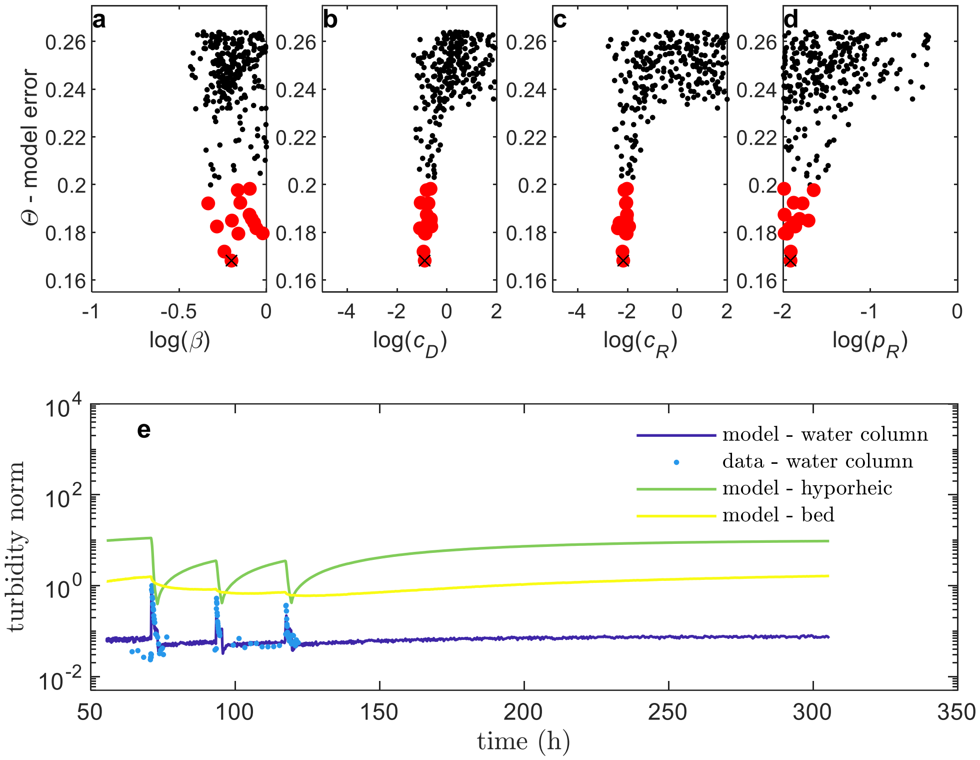

Figure S7. Turbidity Artificial Floods Scenario 2: during stormflow rising and falling limb deposited particles in the hyporheic zone are only allowed to transport back to the water column by setting $p_R = 1$ (Section 2.1.2, Figure 1C). Model error vs. parameter values to demonstrate parameter sensitivity of best-fit model parameters of in-stream measurements of *E. coli* (a-d). Minimum error $\theta = {\sim}0.17$. Best-fit model outputs are plotted against data (e, same as Fig. 2b). *E. coli* norm = concentrations normalized by the maximum concentration in the water column. Black dots represent parameter values that meet a behavioral error threshold (top 1%) and red dots represent optimal parameter values (top 5% of the 1% shown or 0.05% of the total $27 \cdot 10^3$ runs).

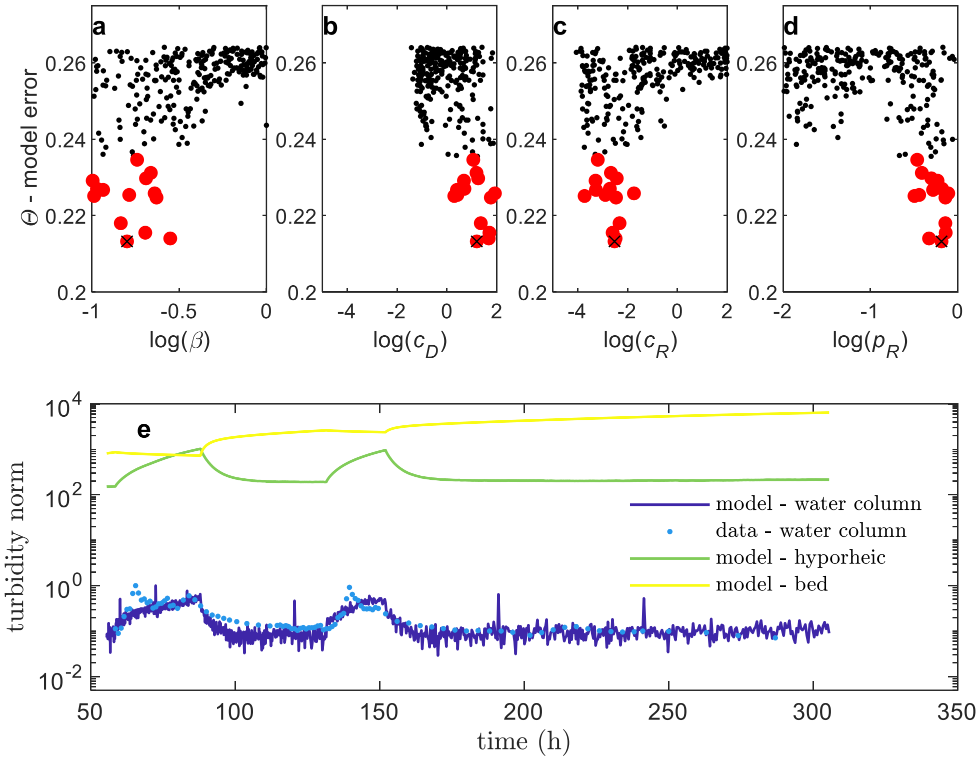

Figure S8. Turbidity natural storm event Scenario 3: during stormflow rising limb deposited particles in the hyporheic zone are only allowed to transport back to the water column by setting $p_R = 1$ (Section 2.1.2, Figure 1E). Model error vs. parameter values to demonstrate parameter sensitivity of best-fit model parameters of in-stream measurements of *E. coli* (a-d). Minimum error $\theta = {\sim}0.21$. Best-fit model outputs are plotted against data (e). *E. coli* norm = concentrations normalized by the maximum concentration in the water column. Black dots represent parameter values that meet a behavioral error threshold (top 1%) and red dots represent optimal parameter values (top 5% of the 1% shown or 0.05% of the total $27 \cdot 10^3$ runs).
